# Cost-effectiveness of cervical cancer screening with primary HPV testing for unvaccinated women in Sweden

**DOI:** 10.1371/journal.pone.0239611

**Published:** 2020-09-30

**Authors:** Sara Fogelberg, Mark S. Clements, Kine Pedersen, Stephen Sy, Pär Sparén, Jane J. Kim, Emily A. Burger

**Affiliations:** 1 Department of Medical Epidemiology and Biostatistics, Karolinska Institutet, Stockholm, Sweden; 2 Department of Health Management and Health Economics, University of Oslo, Oslo, Norway; 3 Center for Health Decision Science, Harvard T. H. Chan School of Public Health, Boston, Massachusetts, United States of America; Rudjer Boskovic Institute, CROATIA

## Abstract

**Background:**

Sweden revised their cervical cancer screening program in 2017 to include cytology-based screening for women aged 23–29 years and primary human papillomavirus (HPV) testing for women aged 30–64 years; however, alternative strategies may be preferred. To inform cervical cancer prevention policies for unvaccinated women, we evaluated the cost-effectiveness of alternative screening strategies, including the current Swedish guidelines.

**Methods:**

We adapted a mathematical simulation model of HPV and cervical cancer to the Swedish context using primary epidemiologic data. We compared the cost-effectiveness of alternative screening strategies that varied by the age to start screening, the age to switch from cytology to HPV testing, HPV strategies not preceded by cytology, screening frequency, and management of HPV-positive/cytology-negative women.

**Results:**

We found that the current Swedish guidelines were more costly and less effective than alternative primary HPV-based strategies. All cost-efficient strategies involved primary HPV testing not preceded by cytology for younger women. Given a cost-effectiveness threshold of €85,619 per quality-adjusted life year gained, the optimal strategy involved 5-yearly primary HPV-based screening for women aged 23–50 years and 10-yearly HPV-based screening for women older than age 50 years.

**Conclusions:**

Primary screening based on HPV alone may be considered for unvaccinated women for those countries with similar HPV burdens.

## Introduction

Cytology-based cervical cancer screening programs have significantly reduced cervical cancer incidence and mortality in most high income countries [1, 2]. Advances in screening technologies provide the opportunity to further improve the effectiveness and efficiency of cervical cancer screening. For example, several clinical studies have shown that human papillomavirus (HPV) testing has a higher sensitivity to detect cervical pre-cancer and cancer than cytology [3–5]. Consequently, several countries, including the United States, Australia, the Netherlands, Italy, Norway and Sweden, have begun to introduce primary HPV testing [6–11]. The introduction of prophylactic HPV vaccines, protecting against two or more of the most oncogenic HPV infections (i.e., HPV16 and HPV18), will also contribute to significant reductions in cervical cancer incidence. Nevertheless, cervical cancer screening will remain the primary preventive measure for the majority of women currently past the age of vaccination eligibility.

In Sweden, national cytology-based screening began in 1973 [12]. The screening program has achieved high coverage with approximately 82% of women complying within six months of the recommended screening intervals [13]. However, cervical cancer remains the third most common cancer in Sweden for women aged 15–44 years [14] and cervical cancer incidence increased 17% between the periods 2002–2013 and 2014–2015 [15]. In 2012, an HPV vaccination program was introduced for females aged 10–12 years, with catch-up vaccination for females through to age 18 years [16]. Moreover, Sweden provided subsidised HPV vaccination for females aged 13–17 years from 2007. In January 2017, primary HPV screening was introduced in the Swedish cervical cancer screening program. The most common HPV test used in Sweden involves partial genotyping for HPV types 16, 18 and 11 other high risk types. The guidelines (herein referred to as the ‘current guidelines’) recommended 3-yearly primary HPV testing for all women aged 30–49 years (implemented as 30–50 years) and 7-yearly primary HPV testing for women aged 50–64 years (implemented as 51–64 years). The recommendations maintained primary cytology-based testing for younger women aged 23–29 years, motivated by the higher prevalence of transient HPV infections among these women [17]. As part of their work in developing the current guidelines, the Swedish National Board of Health and Welfare evaluated the cost-effectiveness of the current program compared to the former cytology-based screening program. Their findings suggested that the current guidelines were less costly and more effective than the former guidelines [17]. However, the published analysis only evaluated these two strategies and did not include a broader range of alternative strategies that may be more effective and more efficient. Including all relevant strategies is a pillar of comprehensive and sound economic evaluations [18].

The cost-effectiveness of cervical cancer screening using primary HPV testing has been previously evaluated in other settings [19]; however, there have been comparatively few cost-effectiveness studies for the Scandinavian countries. In Sweden and Denmark, previous analyses have not included primary HPV screening [20, 21], while one analysis in Norway [22] assessed the cost-effectiveness of introducing primary HPV screening for unvaccinated women. Similar to other studies [19], Burger and colleagues found switching to primary HPV screening was preferred; however, this study did not evaluate primary HPV-based screening for women younger than age 30 years. More recent analyses in Norway have focused on detailed triage interventions [23, 24] or HPV-based screening for fully HPV-immunized women [25].

For unvaccinated populations, primary HPV testing for these women may be considered resource intensive, potentially resulting in more colposcopies due to the higher prevalence of HPV in younger women [26]. However, a recent model-based analysis evaluating the health benefit and resource use trade-offs associated with primary HPV testing strategies for HPV-unvaccinated women concluded that primary HPV testing could start at an earlier age, e.g., at age 25 years, using a more conservative cytology-based triage algorithm for HPV-positive women to control colposcopy use [27]. An Australian study also suggested that primary HPV testing was preferred for unvaccinated women under 30 years of age, as well as for women in vaccinated cohorts [28].

To inform policy-making for cervical cancer screening in Sweden together with countries with similar HPV epidemiologic profiles, our objective was to assess the cost-effectiveness of cervical screening strategies for women not vaccinated against HPV infections within the Swedish context, including an extensive evaluation of primary HPV-based strategies for younger women.

## Materials and methods

### Analytic overview

We adapted an existing mathematical model to reflect the natural history of HPV-induced cervical cancer in Sweden in order to project the health and economic outcomes associated with alternative scenarios of screening. For each screening strategy, we calculated the incremental costs and the incremental health benefits of a strategy compared to the next least costly strategy. We removed strategies that were more costly and less effective (strongly dominated) and strategies that were less costly and less cost-effective (weakly dominated). Effectiveness was represented by quality-adjusted life-expectancy (QALE), which is measured in terms of quality-adjusted life-years (QALYs), incorporating disutility due to cervical cancer [29]. Life expectancy was used as a measure of health benefit in sensitivity analysis. We adopted a societal perspective, and discounted costs and benefits by 3 percent per year, as recommended in Sweden. In line with the cost-effectiveness threshold used by the Swedish National Board of Health and Welfare [17], we considered a strategy with an incremental cost-effectiveness ratio (ICER) below (or within 0.5% of) €85,619 (2014 values) per QALY gained to be cost-effective; an optimal strategy was defined as being close to the cost-effectiveness threshold and having a high acceptability. In addition, we used a similar metric to investigate the trade-off between colposcopy referrals and quality adjusted life-years gained; that is, we calculated an incremental harm-benefit ratio using the ratio of the change in the lifetime expected number of colposcopies per woman divided by the quality adjusted life-years gained [23] of one strategy compared to the next most resource intensive strategy.

### Mathematical simulation model

**Fig 1:**
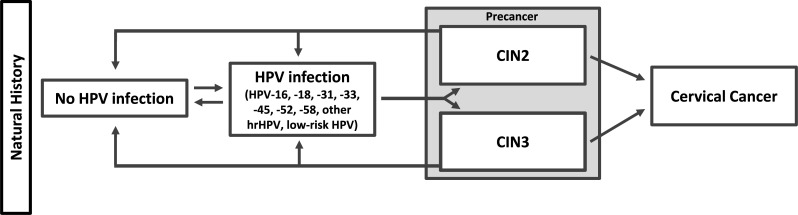
Model schematic for the progression of human papillomavirus (HPV) infection to cervical cancer. HPV infections and pre-cancer (cervical intraepithelial neoplasia (CIN), grade 2 (CIN2) or grade 3 (CIN3)) were stratified by genotype (HPV types 16, 18, 31, 33, 45, 52, and 58; other oncogenic types; and non-oncogenic types). Precancerous health states (CIN2 and CIN3) were considered as heterogeneous entities with differential probabilities of regression and progression to cancer. Progression to cancer required infection with an oncogenic type. Cancer could be symptom-detected at either the local stage, the regional stage, or the distant stage. Women are at risk of dying from background mortality in any state.

The previously developed microsimulation model [30] has been adapted to evaluate cervical cancer prevention strategies in multiple settings, including Norway [22], the United States [31] and low-resource settings [32]. The model simulates a hypothetical cohort of one million individual girls beginning at age 9 years with monthly transitions between health states over the remaining life-time. Health states included healthy (i.e., no HPV infection or cervical abnormality), HPV infection (by HPV genotypes, including types 16, 18, 31, 33, 45, 52, 58, pooled other high-risk HPV types, and low-risk HPV infections), cervical intraepithelial neoplasia (CIN) grades 2 and 3, and squamous cell cervical cancer (by extent of disease, including localized, regional and distant stages). A state chart representation is given in Fig 1. Transition probabilities between health states can be a function of age, HPV type, duration of infection or lesion, and history of prior HPV infection. The transitions between health states include both duration-specific progression and regression and allow for co-infections among seven independent high-risk genotypes (HPV-16,-18, -31, -33, -45 and -58; other high-risk genotypes; and low-risk genotypes). Partial type-specific immunity was modelled by a reduction in the future probability of acquiring an HPV infection following clearance of a previous type-specific infection. We assumed pre-cancerous treatments were successful, such that women with detected and treated cervical pre-cancers returned to a healthy state, although these women may have a raised cervical cancer risk [33]. The probabilities of dying from cervical cancer were based on US stage-specific survival [22], while the probability of dying due to other non-cervical cancer causes reflected female mortality rates in Sweden. The model tracks the resource use and costs associated with screening, diagnosis, management and treatment of cervical HPV infections, pre-cancer and cancer. The model was implemented in C++ with pre- and post-processing in Excel, Stata and R.

We adapted the model to reflect HPV-induced cervical cancer in Sweden using a likelihood calibration approach. The model was run over a grid of input parameters to produce simulation predictions, and then those predictions were compared with the ‘observed’ calibration targets to calculate a log-likelihood. The observed calibration targets included Swedish cancer incidence in a pre-screening population, Swedish age-specific HPV prevalence, HPV type distribution in high grade CIN and in cervical cancer. The input parameter set with the highest log-likelihood was taken as the maximum likelihood estimator and we selected the fifty most likely parameter sets using a likelihood ratio test. We used the 50 best-fitting parameter sets to reflect uncertainty in the underlying natural history of disease. Following the calibration, we validated the model to the Swedish context (see the S1 Material).

### Cervical cancer screening strategies

The current guidelines in Sweden (i.e., from January 2017) recommend triennial cytology-based screening starting at age 23 years and switching at age 30 years to triennial HPV-based testing up to age 50 years, and reducing the screening interval to every seventh year until age 64 years (S1 Fig in S1 File). Management of screen-positive women followed established guidelines [34]. Specifically, women aged 23–29 years who are detected with a low-grade squamous intraepithelial lesion (LSIL) from their primary cytology are reflex tested for high-risk HPV infections, with repeat cytology after six months for women aged 23–27 years and immediate colposcopy for women aged 28–29 years who were HPV positive. Women aged 30 years and older who are high-risk HPV-positive and cytology-negative are followed up after 36 months with direct referral to colposcopy for women who are persistently HPV-positive. At age 41 years, women are provided a one-time co-test with both cytology and an HPV test in order to detect possible HPV-negative CIN or cervical cancers. The previous screening guidelines in Sweden (i.e., until January 2017) involved screening with cytology every three years from age 23 until age 50 years, followed by cytology-based screening every fifth year until age 60 years. For an overview of the current and former screening guidelines evaluated in this analysis, see S1 and S2 Figs in S1 File.

In addition to the current and former guidelines, we evaluated 116 alternative strategies (S3 Table in S1 File). Of these, 54 strategies included variations to the current guidelines, including varying the age at which women switch from primary cytology to primary HPV testing; 54 strategies involved primary HPV testing only (with no preceding cytology); and eight strategies involved cytology only (including the previous Swedish guidelines). For the eight cytology-based strategies, we varied the age to extend the screening interval and the frequency of the screening interval. For the 54 strategies involving a switch from cytology to primary HPV testing, we varied: the age to start screening (ages 23, 26 or 29 years), the age to switch from cytology to HPV testing (ages 25, 30, 35 years), the screening frequency after switching to primary HPV testing (every 3, 5 and 7 years until age 50 years, and every 7 and 10 years for women older than age 50 years), and the wait-time prior to repeat follow-up testing for HPV-positive and cytology-negative women (at 12, 24 and 36 months). For the strategies involving primary HPV screening only, we used similar values to vary the screening start age, screening intervals and follow-up for HPV-positive and cytology-negative women. In order to ensure consistency across the competing strategies, we required all strategies to include a final exit screen at age 60 years or older.

### Assumptions and analyses

An overview of key baseline assumptions and the assumptions for sensitivity analysis are provided in Table 1. In sensitivity analyses, we varied the cost assumptions, diagnostic sensitivity of HPV testing, and compliance to screening and follow-up testing. To explore the impact of alternative costing assumptions, we replaced our base-case assumptions that used cost estimates from Östensson et al [35] with estimated costs from the National Board of Health and Welfare [17]. Compared with the base-case costs, the costs from the National Board of Health and Welfare were lower for screening and for treatment of localized cancer. Both sets of costs assumed that the cytology and HPV tests were the same cost.

**Table 1:** Selected model inputs for key assumptions in base case analysis and sensitivity analyses.

	Base case	Cost assumptions sensitivity analysis	HPV-test sensitivity analysis (cervical cancer)	HPV-test sensitivity analysis (other health states)	Compliance assumptions sensitivity analysis
***Costs* (€)**					
**Screening with cytology/HPV-test**	111	71	111	111	111
**Colposcopy** with biopsy	318	431	318	318	318
**Treatment for CIN**	410	410	410	410	410
**Cancer treatment (localized)**	27580	15254	27580	27580	27580
**Cancer treatment (regional)**	52775	56859	52775	52775	52775
**Cancer treatment (distant)**	62925	78461	62925	62925	62925
***Utilities (duration of five years)***					
**Cancer diagnosis (localised)**	0.76	0.76	0.76	0.76	0.76
**Cancer diagnosis (regional)**	0.67	0.67	0.67	0.67	0.67
**Cancer diagnosis (distant)**	0.48	0.48	0.48	0.48	0.48
***Test characteristics***					
**Pro**bability of HR-HPV given CA *	100%	100%	90%	90%	100%
**Prob**ability of HR-HPV given HR-HPV (other health states)	100%	100%	100%	90%	100%
***Compliance***					
**Compliance screening**	100%	100%	100%	100%	80%
**Compliance follow-up for HPV+/cyt- women**	100%	100%	100%	100%	90%
**Compliance colposcopy**	100%	100%	100%	100%	95%

Notes: Costs for screening, colposcopy, and treatments include patient time and office costs. Costs were valued in 2014-SEK and converted to EUR (€ 1 = SEK 9.099). Baseline assumptions for costs from Östensson et al (2015); cost assumptions for the cost sensitivity analysis are from the National Board of Health and Welfare (2015). Abbreviations: CA, cancer; HPV human papillomavirus; HPV+/Cyt-, HPV-positive, cytology-negative result.

*Given cervical cancer, a proportion of women will test positive (or negative) for HR-HPV and subsequent tests for that woman will be the same.

The sensitivity of HPV testing (defined as the probability of HPV DNA-positive given an HPV DNA infection is present) was assumed to be 100 percent in our base case analysis. Due to the growing literature on HPV-negative cervical cancer tumours [36–38], we varied this assumption in sensitivity analyses by 1) assuming an HPV test sensitivity of 90 percent for women with cervical cancer, and 2) assuming an HPV test sensitivity of 90 percent for all women (with or without cervical cancer). HPV specificity (defined at the infection level as the probability of being test negative to any high-risk HPV given no high-risk HPV infection) is assumed to be 100% in both the base-case and sensitivity analyses. Compliance with screening, follow-up testing and colposcopy with biopsy was assumed to be 100 percent in our base case analysis. To justify the base case assumption of 100 percent compliance, future screening behaviour is highly uncertain, and may change as the screening strategy changes and this value supports comparisons with previous publications. In sensitivity analyses, we assumed imperfect screening compliance including (i) a screening compliance of 80 percent, (ii) follow-up compliance of 90 percent, and (iii) a colposcopy compliance of 95 percent, in line with Swedish screening registries [13]. We also included a sensitivity analysis where we replaced QALYs with life-years. Finally, we conducted a probabilistic sensitivity analysis on the natural history parameter values to identify the variation in the cost-efficiency frontier under parameter uncertainty.

## Results

### Primary analysis

**Fig 2:**
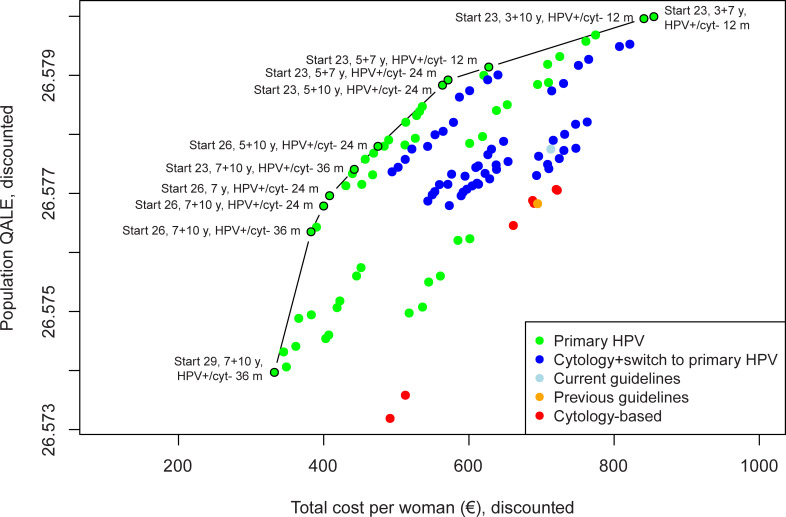
Discounted (3%) cost-effectiveness of 116 screening strategies under the baseline assumptions, including the 11 strategies on the cost-effectiveness efficiency frontier (black circles). The no screening strategy (natural history) has been excluded from the graph. Strategy nomenclature: “Start” indicates screening start age, y indicates years (before and after age switch), and human papillomavirus (HPV)+/cyt- m indicates the follow-up time for HPV-positive, cytology negative women (in months).

Both the current (HPV-based after age 30 years) and previous (cytology-based for all ages) screening guidelines in Sweden were more costly and less effective than alternative screening strategies (Fig 2). The preferred strategies involved primary HPV testing for women of all screening target ages, which dominated strategies involving primary cytology (with or without switching to primary HPV testing). Specifically, out of the 116 evaluated strategies, we identified 11 HPV-based strategies as efficient with ICERs ranging from €2,986 to €401,441 per QALY gained (Table 2). For these strategies, the lifetime cancer risk ranged from 0.13 to 0.30 percent, the proportion of cancers detected at a local stage ranged from 65.1 percent to 70.0 percent, and the number of colposcopy referrals per woman over their lifetime ranged from 0.34 to 1.33.

**Table 2:** Analytic outcomes for the no intervention scenario, the current and former Swedish guidelines, and strategies on the cost-efficiency frontier under the baseline assumptions.

*Screening strategy characteristics*						*Model predictions*						
**Screening regime**	**Cytology-based screening**	**Screening start age (years)**	**Screening interval (years) before age 50**	**Screening interval (years) after age 50**	**HPV-pos/Cyt-neg follow-up (months)**	**Expect no of colposcopy referrals per woman over lifetime**	**Lifetime cervical cancer risk (%)**	**Proportion of cancers detected at local stage (%)**	**Total cost per woman (discounted) (€)**	**Discounted QALE**	**Undiscounted life-years (from age 9 years)**	**ICER (Cost (€) per QALY gained)**
Natural History							1.73	51.0	210	26.53298	74.99756	
Previous program	Yes	23	3	5	-	0.48	0.28	64.6	695	26.57683	75.22759	Dominated
Current program	Before age 30	23	3	7	36	0.67	0.19	70.0	713	26.57775	75.23537	Dominated
Primary HPV only	No	29	7	10	36	0.34	0.30	67.6	332	26.57397	75.22387	2986
Primary HPV only	No	26	7	10	36	0.43	0.24	66.8	383	26.57635	75.22958	21190
Primary HPV only	No	26	7	10	24	0.52	0.23	67.4	400	26.57679	75.23065	39748
Primary HPV only	No	26	7	7	24	0.53	0.22	68.4	408	26.57696	75.23217	46790
Primary HPV only	No	23	7	10	36	0.53	0.22	65.5	442	26.57741	75.23196	76163
Primary HPV only	No	26	5	10	24	0.60	0.20	68.3	475	26.57780	75.23545	83728
Primary HPV only	No	23	5	10	24	0.76	0.17	68.5	564	26.57883	75.23748	85736
Primary HPV only	No	23	5	7	24	0.77	0.17	69.1	571	26.57892	75.23806	87994
Primary HPV only	No	23	5	7	12	1.04	0.16	67.1	627	26.57914	75.23861	257471
Primary HPV only	No	23	3	10	12	1.31	0.13	66.1	841	26.57996	75.24110	260483
Primary HPV only	No	23	3	7	12	1.33	0.13	65.1	855	26.57999	75.24120	401441

The highlighted row represents the optimal strategy under the Swedish cost-effectiveness threshold.

Abbreviations: CA, cancer; HPV, human papillomavirus; HPV-pos/Cyt-neg, HPV-positive, cytology-negative; QALE, quality-adjusted life expectancy; QALY, quality-adjusted life-year; ICER, incremental cost-effectiveness ratio.

Given the Swedish cost-effectiveness threshold of €85,619 per QALY gained, the optimal strategy involved primary HPV testing starting at age 23 years with 5-yearly screening intervals until age 50 years, followed by 10-yearly screening until an exit test after age 60 years (Fig 2). The optimal strategy involved women who were HPV-positive and cytology-negative at their primary screen (for women of all ages) receiving repeat testing at 24 months, rather than waiting 36 months as recommended by the current guidelines. Assuming perfect compliance, the optimal strategy was associated with a lifetime risk of developing cervical cancer of 0.17 percent, 0.76 colposcopy referrals per woman over their lifetime and a discounted lifetime cost of €564 per woman. In comparison, the current Swedish screening program was projected to have a higher lifetime risk of cervical cancer (0.19 percent), and a higher discounted lifetime cost per woman (€713), although fewer colposcopy referrals (0.67 per women over their lifetime). Similarly, the former cytology-based Swedish screening program also had a higher lifetime risk (0.28 percent), fewer colposcopies (0.48 per woman over their lifetime), and a higher discounted cost per woman (€695) compared to the optimal strategy. More intensive screening strategies than the optimal strategy were generally associated with a higher proportion of women diagnosed with cervical cancer at an earlier stage (Table 2). For example, the optimal strategy was projected to detect 68.5 percent of cancer cases at a local stage, compared with 70 percent for the current guidelines. The previous cytology-based screening guidelines were associated with 64.6 percent local cancers.

**Fig 3:**
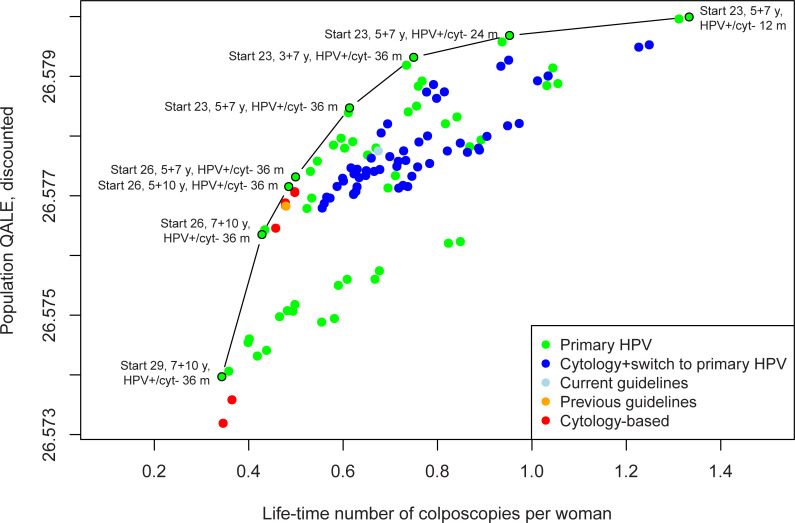
Discounted (3%) quality-adjusted life expectancy and lifetime number of colposcopy referrals per woman for the 116 screening strategies under the base case assumptions, including the eight strategies on the colposcopy referral per QALY gained frontier. The no screening strategy (natural history) has been excluded from the graph. Strategy nomenclature: Start indicates start age, y indicates years (before and after age switch) and HPV+/cyt- m indicates the follow-up time for HPV-positive, cytology negative women (in months).

Similar to when using the cost per QALY gained efficiency metric, all efficient strategies involved primary HPV testing when reporting outcomes of colposcopies and QALEs (Fig 3, Table 3). For these strategies, the number of colposcopy referrals per woman over her lifetime ranged from 0.34 to 1.33, and the lifetime cancer risk ranged from 0.13 to 0.30 percent.

**Table 3:** Summary of the colposcopy-efficiency frontier under the baseline assumptions.

*Screening strategy characteristics*						*Model predictions*					
**Screening regime**	**Cytology-based screening**	**Screening start age (years)**	**Screening interval (years) before age 50**	**Screening interval (years) after age 50**	**HPV-pos/Cyt-neg follow-up (months)**	**Lifetime CA risk (%)**	**Proportion of localized CA (%)**	**Expect no of colposcopy referrals per woman over lifetime**	**Total costs (discounted)€**	**Discounted QALE**	**Colposcopy referrals per QALY gained**
Natural History						1.73	51.0		210	26.53298	
Previous program	Yes	23	3	5	-	0.28	64.6	0.48	695	26.57683	Dominated
Current program	Before age 30	23	3	7	36	0.19	70.0	0.68	713	26.57775	Dominated
Primary HPV	No	29	7	10	36	0.30	67.6	0.34	332	26.57397	8.36740
Primary HPV	No	26	7	10	36	0.24	66.8	0.43	383	26.57635	35.72655
Primary HPV	No	26	5	10	36	0.22	68.1	0.49	452	26.57715	70.89125
Primary HPV	No	26	5	7	36	0.20	69.5	0.50	467	26.57731	90.73312
Primary HPV	No	23	5	7	36	0.18	68.2	0.61	536	26.57847	98.54284
Primary HPV	No	23	3	7	36	0.14	68.8	0.75	725	26.57932	159.9218
Primary HPV	No	23	3	7	24	0.14	67.0	0.95	775	26.57968	564.2489
Primary HPV	No	23	3	7	12	0.13	65.1	1.33	855	26.57999	1227.413

Abbreviations: CA, cancer; HPV, human papillomavirus; HPV-pos/Cyt-neg, HPV-positive, cytology-negative; QALE, quality-adjusted life expectancy; QALY, quality-adjusted life-year.

### Sensitivity analyses

Primary HPV-based strategies at 5-yearly intervals starting at age 23 years remained optimal across all sensitivity analyses, but the optimal screening frequency for women aged 50 years and older as well as the wait-time prior to repeat testing for HPV positive, cytology negative women varied across sensitivity analyses (S4–S7 Tables and S7 Fig in S1 File). For example, when we assumed a lower cost of screening and treatment procedures, the optimal strategy involved screening more frequently (i.e., 7-yearly intervals) after age 50 years, and a longer wait (36-month) for HPV-positive, cytology-negative women. Similarly, in the scenarios where we assumed a lower sensitivity of the HPV test and imperfect compliance, the optimal strategy also involved 7-yearly screening after age 50 years, but with a 24-month follow-up wait-time for HPV-positive, cytology-negative women. Finally, strategies involving primary HPV-based screening remained less costly and more effective than strategies involving cytology when we varied the assumptions concerning screening behaviour, HPV test characteristics and costs. For example, varying the HPV test sensitivity for cancer had minor impact on the rank order of the strategies and did not change the optimal strategy compared with the base-case analysis.

When we estimated the proportion of the parameter sets where strategies were on the efficiency frontier (probabilistic sensitivity analysis), we found that the strategy involving 5-yearly screening with primary HPV starting at age 23, 7-yearly screening from age 50, and 24 months follow-up time for HPV-positive, cytology-negative women was consistently projected to be on the efficiency frontier in 94 percent of the parameter sets (S8 Table in S1 File). However, given the Swedish cost-effectiveness threshold, this strategy was considered optimal in only 6 percent of the parameter sets. The strategy considered optimal in the base-case analysis (involving 5-yearly screening with primary HPV starting at age 23, 10-yearly screening from age 50, and 24 months follow-up time for HPV-positive but cytology-negative women), was on the efficiency frontier in 36 percent of the sets, and was optimal considering the cost-effectiveness threshold in 12 percent of the sets. The strategy that was optimal in the cost assumption sensitivity analysis was on the frontier in 24 percent of the parameter sets, and optimal considering the willingness to pay threshold in 18 percent of the parameter sets.

Importantly, strategies involving primary HPV testing for women of all ages were optimal across all 50 parameter sets. In general, 5- and 7- yearly screening for women younger than age 50 years were optimal in 62 and 38 percent of the sets, respectively. For women aged over 50 years, 10-yearly and 7-yearly screening were optimal in 68 and 32 percent of the sets, respectively. Follow-up time for HPV-positive, cytology-negative at 12, 24 and 36 months were optimal in 10, 66 and 24 percent of the sets, respectively. A start age of 23 and 26 years were optimal in 54 and 46 percent of the sets, respectively. In sum, consistency across the parameter sets supported primary HPV starting at age 23 years with 5-yearly screening before age 50 years and 10-yearly screening after age 50 years, with a follow-up for HPV-positive, cytology-negative women at 24 months.

## Discussion

Using a natural history model of HPV-induced cervical cancer contextualized with primary epidemiologic data from Sweden, we explored a comprehensive set of screening strategies and found that primary HPV-based screening alone was preferred over both cytology-based strategies and strategies that involved primary HPV screening preceded by cytology. Given the Swedish cost-effectiveness threshold of €85,619 per QALY gained, the optimal screening strategy in our analysis involved primary HPV testing starting at age 23 years, with 5-yearly screening until age 50 years followed by 10-yearly screening until a single exit screen after age 60 years. The optimal strategy also involved that women testing HPV-positive and cytology-negative are offered a repeat HPV test in 24 months with referral to colposcopy for women with a persistent HPV infection. Compared to the recently implemented guidelines in Sweden, the optimal strategy identified in our analysis unifies the screening program with a single primary test, requires less frequent primary screening intervals (with more intensive follow-up of HPV-positive, cytology-negative women), and provides more health benefit at a lower cost. Note that introducing self-sampling, as a complement to office-based collection, and a reduction in costs due to testing at scale may further improve the cost-effectiveness of primary HPV testing.

In our sensitivity analyses, primary HPV testing for all ages remained consistently more effective and less costly, the optimal start age remained at 23 years, and 5-yearly screening intervals for women under 50 remained optimal. Assuming either 90 percent sensitivity for HPV tests, or changing compliance or cost assumptions resulted in a strategy that reduced the screening interval to seven years for women over age 50 years compared to the optimal strategy identified in the base case analysis. Importantly, the optimal strategy from the base case analysis was identified as cost-efficient in 36 percent of the parameter sets, while the corresponding strategy with seven-year intervals for women over 50 years of age was on the efficiency frontier for 94% of the parameter sets.

Overtreatment and unnecessary colposcopies are a potential concern when using primary HPV testing for women less than age 30 years. When using colposcopy as an outcome, we found that primary HPV screening alone continued to dominate the other strategies. An important difference compared to the cost-effectiveness analysis was that the majority of the strategies on the colposcopy frontier involved waiting 36 months for HPV-positive and cytology negative women rather than 24 months. There is, however, a trade-off between follow-up time for colposcopy and effectiveness, where longer follow-up intervals for colposcopy may reduce resource use and costs by allowing for HPV to spontaneously clear, while possibly leading to disease progression and poorer outcomes (Table 3).

The cost-effectiveness of primary HPV screening in women under 30 years of age may reflect country-specific variations in HPV prevalence, which in Sweden is lower compared to Denmark and Norway [12]. However, the transient nature of HPV infections in younger women coupled with a longer follow-up time (such as 24–36 months) may mitigate the potential for overtreatment, even in populations where the HPV prevalence is higher than in Sweden.

A strong advantage of our modelling approach is that we calibrated the model to detailed, linked health and population registers from Sweden. Using the calibrated model, we were able to validate the model predictions with data that were not used to inform the model inputs; from the validation, we found strong consistency with observed Swedish data for contemporary cervical cancer incidence rates (see S1 Material for further details). We also evaluated a broad range of candidate strategies not previously evaluated in any cost-effectiveness analyses for unvaccinated women. Importantly, a previous cost-effectiveness analysis conducted by the Swedish National Board of Health and Welfare only investigated a narrow set of screening scenarios, which may have resulted in adoption of inefficient national guidelines. This analysis highlights the importance of considering a more comprehensive set of strategies, such as primary HPV testing for all ages. Finally, we re-calculated the results under parameter uncertainty and under several sensitivity analyses, confirming our base case results.

Limitations include, firstly, the potential validity of the calibration targets, the underlying natural history model and the restriction of the natural history model to squamous cell carcinoma. The exclusion of adenocarcinoma from the model is expected to lead to conservative predictions for the benefits due to primary HPV testing [5]. Although the model assumes that all cervical cancers are due to HPV infection, studies conducting HPV typing of invasive cervical cancer tends to confirm less than 100% of invasive cervical cancer to include high-risk HPV. High-risk HPV negative cancers may reflect a separate causal pathway or lack of high-risk HPV detectability at cancer diagnosis. To address this potential limitation, we varied the sensitivity of HPV testing to detect cervical cancer and found that the optimal strategy under this assumption did not change. Finally, there are limited data available on utilities for cervical cancer screening. We partially address this limitation by a sensitivity analysis using discounted life-years as the measure of effectiveness.

In 2019, the National Working Group for Cervical Cancer Prevention issued a recommendation for partial HPV genotype-specific management for women who are HPV 16- or 18-positive and with a normal reflex cytology test. These new recommendations involved a more intensive follow-up, i.e., a new HPV test in either 1 or 5 years rather than the usual 3 or 7 years, for the two most carcinogenic HPV types. The cost-effectiveness of this new recommendation has not been assessed, but should be evaluated in future analyses.

Our analysis focused on cohorts of women not vaccinated against HPV infections. In the near future, it is important to evaluate the cost-effectiveness of screening strategies in the context of HPV vaccination, which is particularly relevant for birth cohorts who are about to initiate screening. Recent studies have suggested that screening for HPV-vaccinated women should involve primary HPV testing at a lower screening frequency than for unvaccinated women [25, 31]. However, most of the women being currently screened are from unvaccinated cohorts, which will continue to be an important screening population for many decades to come.

## Conclusions

In conclusion, our model-based analysis supports a universal primary HPV screening program in Sweden, including women less than age 30 years, for unvaccinated cohorts, which may also be applicable in countries with similar HPV burden and health care systems. In particular, we found that cervical cancer screening starting at age 30 years, as implemented in some European countries [7, 39], was suboptimal for the Swedish context. Strategies involving primary HPV for starting at age 23 years dominated in both the cost-effectiveness analyses as well as in our benefit-harm trade-off analysis (colposcopy referrals per QALY gained).

## Supporting information

S1 File (PDF)

S1 Material (ZIP)
