# Supplementary material for: Cost-effectiveness of cervical cancer screening with primary HPV testing for unvaccinated women in Sweden
Source: PLoS One. 2020 Sep 30;15(9):e0239611. doi: 10.1371/journal.pone.0239611 (PMC7526933; doi:10.1371/journal.pone.0239611)
Supplement: S1 File — (PDF) [file pone.0239611.s001.pdf]

Supplementary Material

Model and parameterization

The results in this paper were generated by a first-order Monte Carlo simulation model (see Campos et al 2014, Kim et al 2015, Burger et al 2017). The model simulated individual women starting at age 9 years until death and followed them through a series of mutually exclusive, collectively exhaustive health states. Model outputs included lifetime cancer risk, quality-adjusted life-years, cervical cancer mortality and costs. The model simulated for one million women for each intervention.

The model calibration (or 'model fitting') was undertaken with a set of calibration targets based on unscreened populations. The model was run over a grid of input parameters to produce simulation predictions, and then those predictions were compared with the 'observed' calibration targets to calculate a log-likelihood. The input parameter set with the highest log-likelihood was taken as the maximum likelihood estimator and we selected the fifty most likely parameter sets using a likelihood ratio test.

We assumed that the mathematical representation for the natural history model was the same between populations. Further, it was assumed that a subset of the natural history parameters were the same between populations, including transition probabilities from HPV infection to disease. These parameters were taken from the published literature. Finally, we calibrated specific parameters to the calibration targets using primary data from Sweden.

Cost assumptions

Our data on costing were taken from Östensson et al (2015). We ran the model using Swedish kronor (SEK) using estimates from Östensson et al reported in Euros with a reported exchange rate of 10.62 SEK to 1 Euro. Values were then inflated to 2014 years value using a consumer price index from Statistics Sweden[1]. Values reported in this paper were then converted from SEK to an average of the conversion rate between SEK and Euros for 2014 (9.099 Swedish kronor for 1 Euro[2]). We considered both direct and indirect costs in the model, where direct costs included test supplies, staff time and office costs. Indirect costs were defined as a woman's missed income from having participated in screening, colposcopy, treatment for CIN or treatment for cancer. Income per hour was defined as the hourly average wage in Sweden 2014. Transportation costs were not included in this analysis. Costs for starting up the screening strategies were not included, so that the screening strategies are compared under the assumption of steady-state conditions (Husereau et al 2013).

Östensson et al reported costs for stages FIGO Ia1-Ib1, FIGO Ib2, FIGO II, FIGO III and FIGO IV. We used primary data to calculate weights used to fit the costing data to the categories for local, regional and distant cancer. In Östensson et al, the cost of a Pap-smear/HPV test were

[1] http://www.scb.se/hitta-statistik/statistik-efter-amne/priser-och-konsumtion/konsumentprisindex/konsumentprisindex-kpi/

[2] https://www.ecb.europa.eu/stats/policy_and_exchange_rates/euro_reference_exchange_rates/html/eurofxref-graph-sek.en.html

reported together with the office costs associated with such a test (including the cost of a clinical room and wage for a midwife). In order to separate between test costs and office costs, we used a cost estimate for self-sampled HPV-tests from a working paper by Östensson et al [3].

Table S1. Costing data for the base case analysis.

Category	SEK (2014)	EUR (2014)	Description
Pap-smear	233	25.6	Cost of material, lab procedure (including wage costs) and lab material.
HPV test	233	25.6	Cost of material, lab procedure (including wage costs) and lab material.
Office cost for pap-smear/HPV test	491	54.0	Wage cost, cost of clinic room.
Patient time for pap-smear/HPV test	289	31.8	Based on the hourly average wage of 30.4 Euros
Office cost for colposcopy with biopsy	2538.62	279.0	Cost of material, wage costs, lab costs, cost of clinic room.
Patient time for colposcopy with biopsy	356.3	39.2	Based on the hourly average wage of 30.4 Euros
Treatment of CIN	3730	410.0	Cost of material, wage costs, lab costs, cost of clinic room. Includes cost of patient time.
Treatment of local cervical cancer	250931.7	27579.5	Costs of treating FIGO Ia1-Ib1 (81%) and FIGO 1b2 (19%). Cost of surgery, radiation, chemotherapy, wages, patient time for treatment.
Treatment regional cervical cancer	480171.8	52774.8	Costs of treating FIGO II (50%) and FIGO II (50%). Cost of surgery, radiation, chemotherapy, wages, patient time for treatment.
Treatment distant cervical cancer	572525	62925.2	Costs of treating FIGO IIII. Cost of surgery, radiation, chemotherapy, wages, patient time for treatment.

Abbreviations: SEK Swedish kronor; EUR Euros; HPV human papillomavirus; CIN cervical intra-epithelial neoplasia; FIGO International Federation of Gynecology and Obstetrics.

Costs for our sensitivity analysis were taken from a cost-benefit analysis by Socialstyrelsen (2015).

[3] Östensson et al. "Cost-effectiveness of organized primary Human papilloma virus (HPV) testing on a HPV vaccinated population" Working paper.

Utilities

Age-specific background utilities were based on data from Denmark (Olsen and Jepsen 2010; see Table S2). For those diagnosed with cervical cancer, an overall utility was calculated by the background utility multiplied by a stage-specific utility for a five-year period (see Table 1).

Table S2: Age-specific background utilities, Denmark.

	Age group (years)								
	0-	20-	30-	40-	50-	60-	70-	80-	100-
Utility	1	0.9203	0.9118	0.8763	0.8499	0.8552	0.832	0.6919	0.6919

Strategies and assumptions

In our base case analysis, we varied previous cytology based strategies by alternating the switch age to longer screening intervals, and screening intervals for older women. For the revised, current guidelines that used a mix of cytology and primary HPV testing, we varied the start age, switch age to primary HPV, screening intervals for women over 50 and follow-up time for HPV positive and cytology negative women. Finally, we varied the start age, screening interval and screening interval for women over 50 years of age and follow-up for HPV positive and cytology negative women for a set of primary HPV screening strategies.

We made the following assumptions for all strategies: 1) all women with detected CIN 2 or CIN 3 were treated (i.e. no wait and follow-up period for younger women with CIN; 2), all HPV positive and LSIL women were referred to immediate colposcopy/biopsy except women under 28 years of age (who were referred to repeat cytology after 6 months); 3) treatment was assumed to be 100 percent effective; 4) treatment was followed up with cytology and HPV triage after six months and then again after six months; and 5) HPV positive women with colposcopy/biopsy confirmed CIN 1 were not treated but followed up with new colposcopies/biopsies after 12 and 24 months.

In strategies with a switch to primary HPV testing, or with primary HPV testing only, women were assumed to have one co-test with cytology after age 40 years. This reflected the decision to include a co-test at age 41 in the current and revised screening guidelines.

Table S3. A summary of the evaluated screening strategies, with screening regimes defined by combinations of the column values.

Screening regime	Screening start age	Age to switch to primary HPV	Primary HPV screening interval	Age to extend screening interval for older women	Extended screening interval for older women	Repeat screen for HPV+/cyt - women (months)
Previous cytology based guidelines	23	—	—	50	5	—
-Variations	—	—	—	45, 50, 55	5, 7	—
Current, revised, guidelines	23	30	3	50	7	36

Variations with preceding cytology	—	25, 30, 35	3, 5, 7	50	7, 10	12/24/36
Variations with no preceding cytology (primary HPV screening only)	23, 26, 29	—	3, 5, 7	50	7, 10	12/24/36

Abbreviations: HPV human papillomavirus; HPV+/Cyt- HPV-positive, cytology-negative.

Screening strategies

Figure S1. Current national Swedish guidelines for cervical cancer screening (implemented spring 2017).

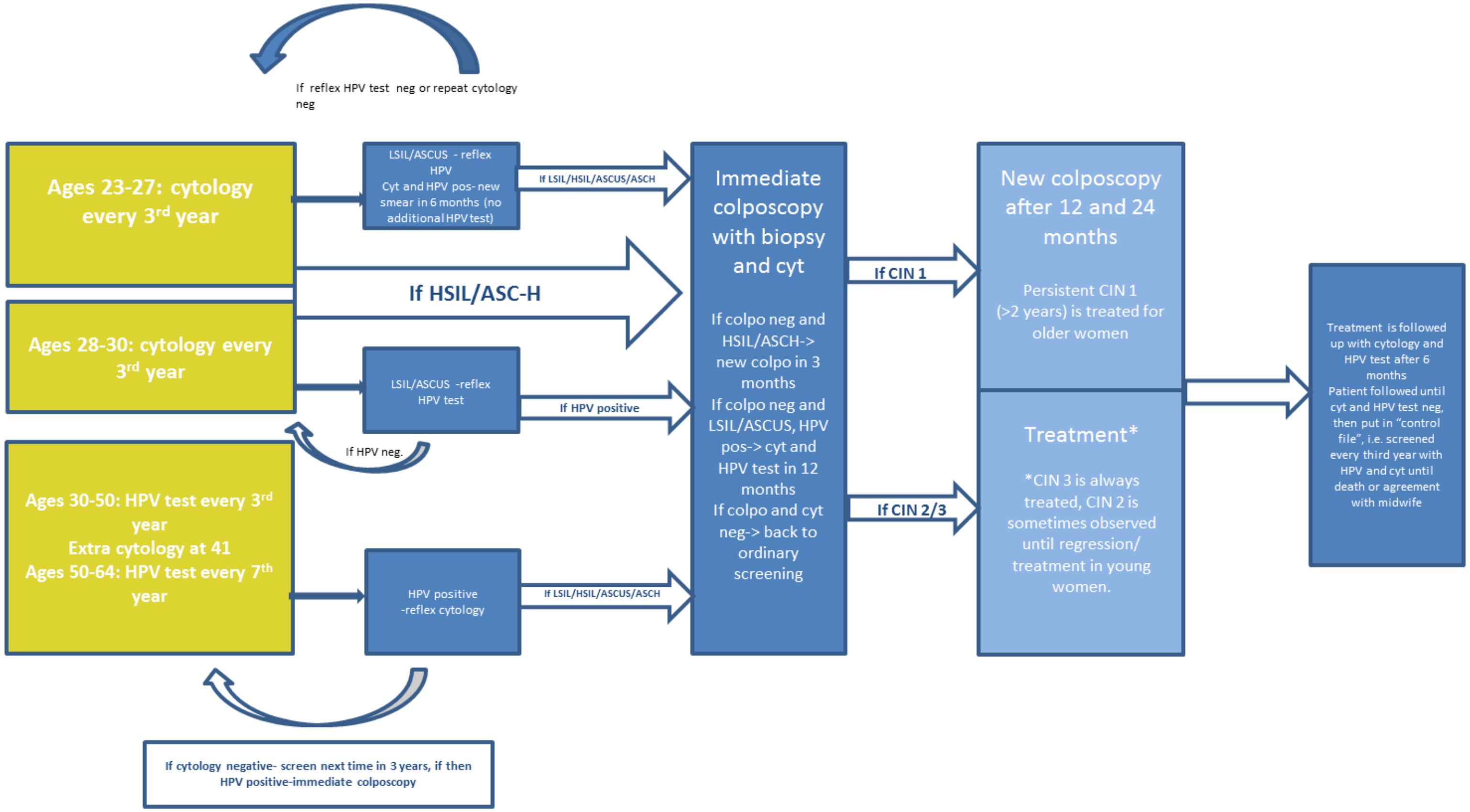

Figure S2. Previous cytology based national Swedish guidelines (in use between 1973 and 2016)

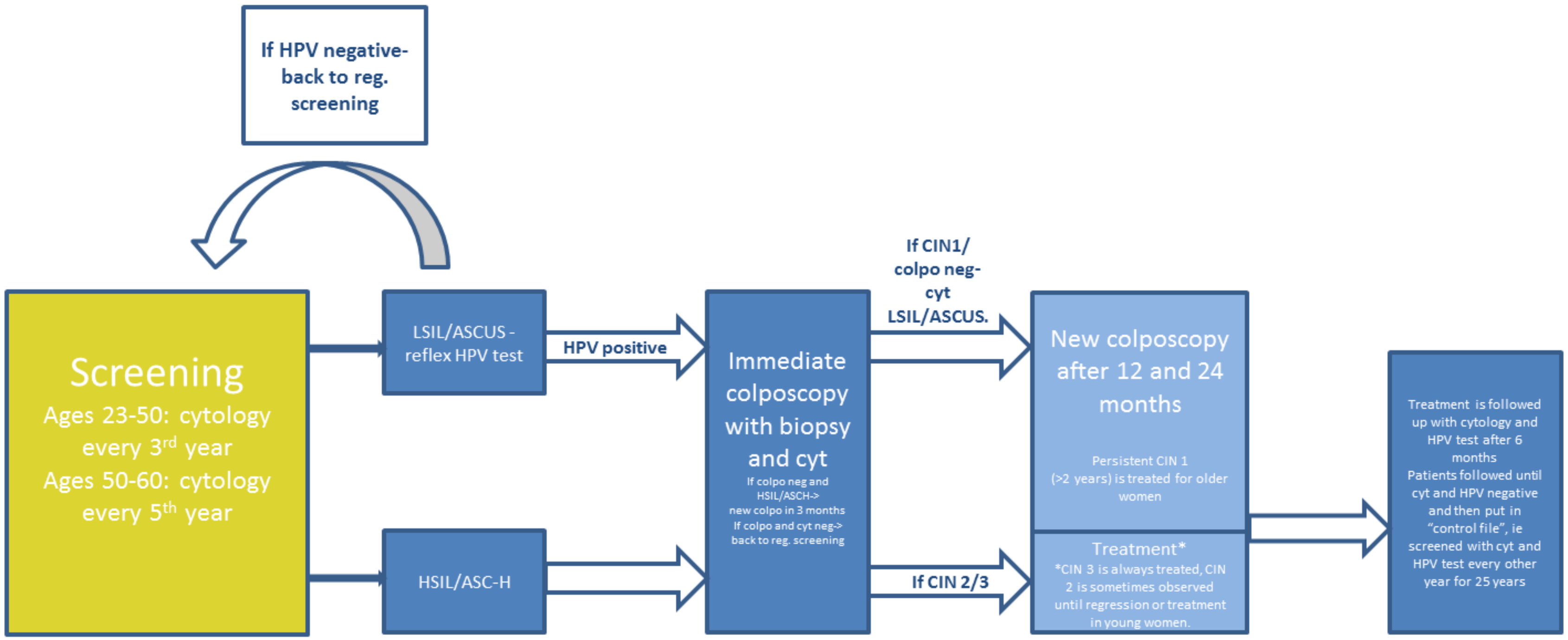

Calibration targets

The Swedish calibration targets included (i) age-specific HPV prevalence for HPV 16, 18, 31, 33, 45, 52 and 58, (ii) the HPV type distribution in high grade CIN and (iii) the HPV type distribution in cervical cancer. For each point estimate, we determined the proportion with a binomial confidence interval using primary data from 1980-2002. Cancer incidence bounds were generated using the maximum and minimum age-specific annual incidence for the years 1958-1969.

Validation and model fitting

The HPV prevalence data for healthy women, women with CIN and women with cervical cancer were described by Pedersen et al. (2017). See below for some of the validation results.

Figure S3: Simulated HPV 16 prevalence in healthy women from 50 good fitting sets, with upper and lower bounds for women in Sweden.

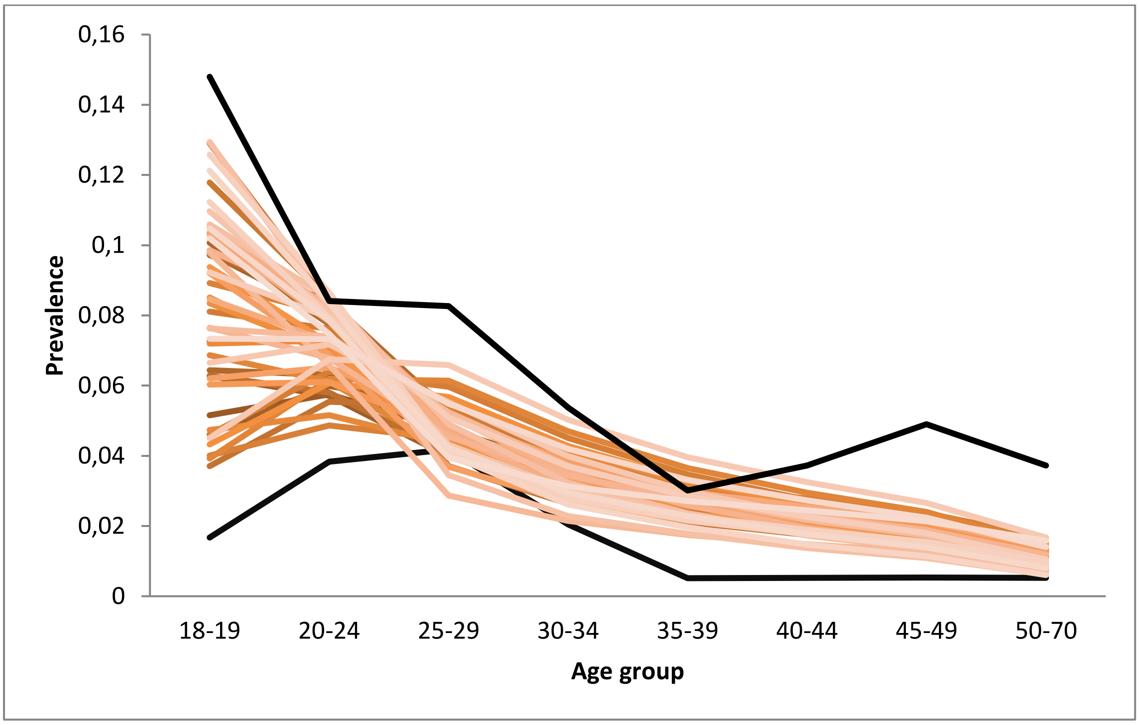

Figure S4: Violin plots of the simulated HPV prevalence in women with CIN 3 from 50 good fitting sets and the upper and lower bounds for women in Sweden.

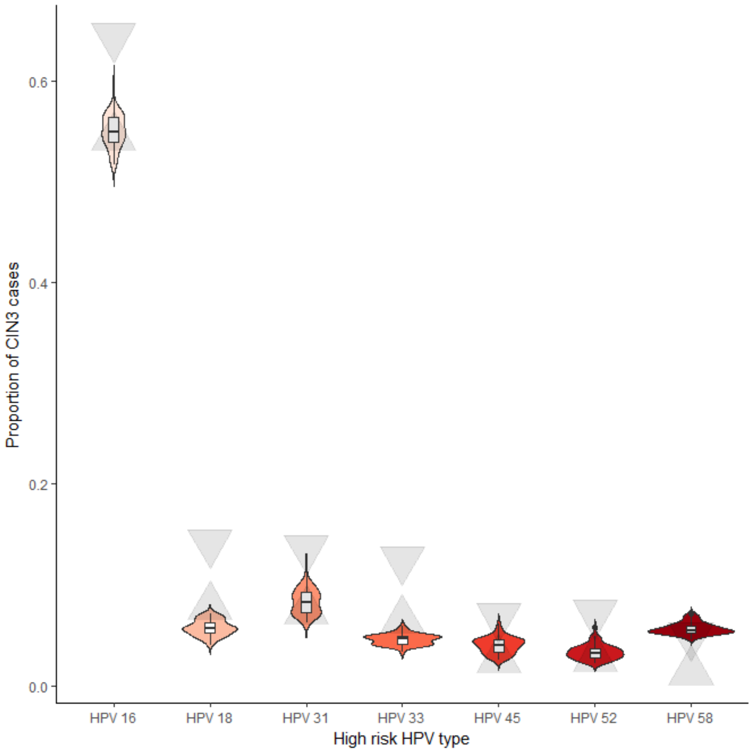

Figure S5: Violin plots of the simulated HPV prevalence in women with cervical cancer from 50 good fitting sets and the upper and lower bounds for women in Sweden.

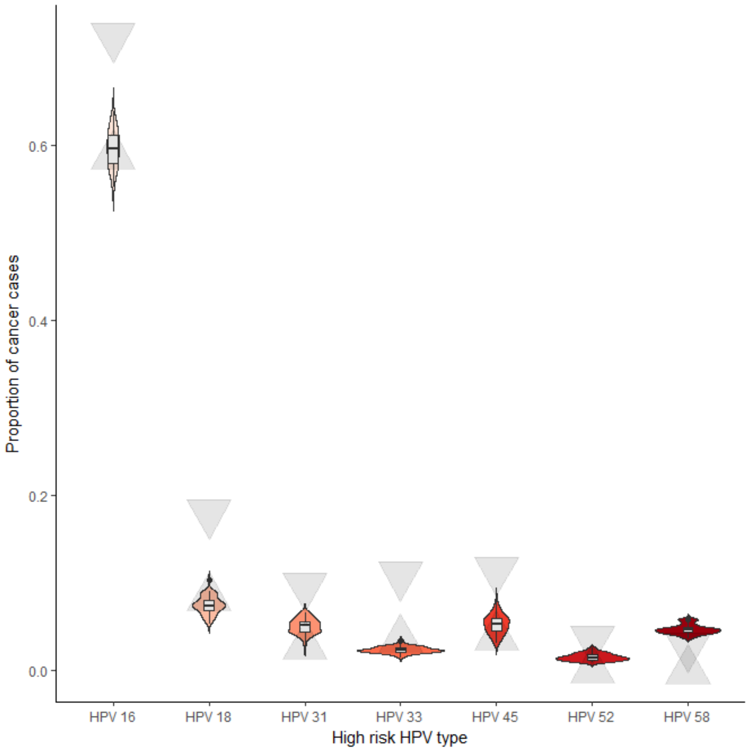

We also validated the model using cervical cancer data for 1958-1969, i.e. before there was a national cervical cancer screening program in Sweden. The primary data source was described in Sparén (1996). The empirical boundaries were the minimum and maximum values over the defined years for the specific age groups.

Figure S6: Simulated cervical cancer incidence from 50 good fitting sets and the upper and lower bounds from women in Sweden 1958-1969.

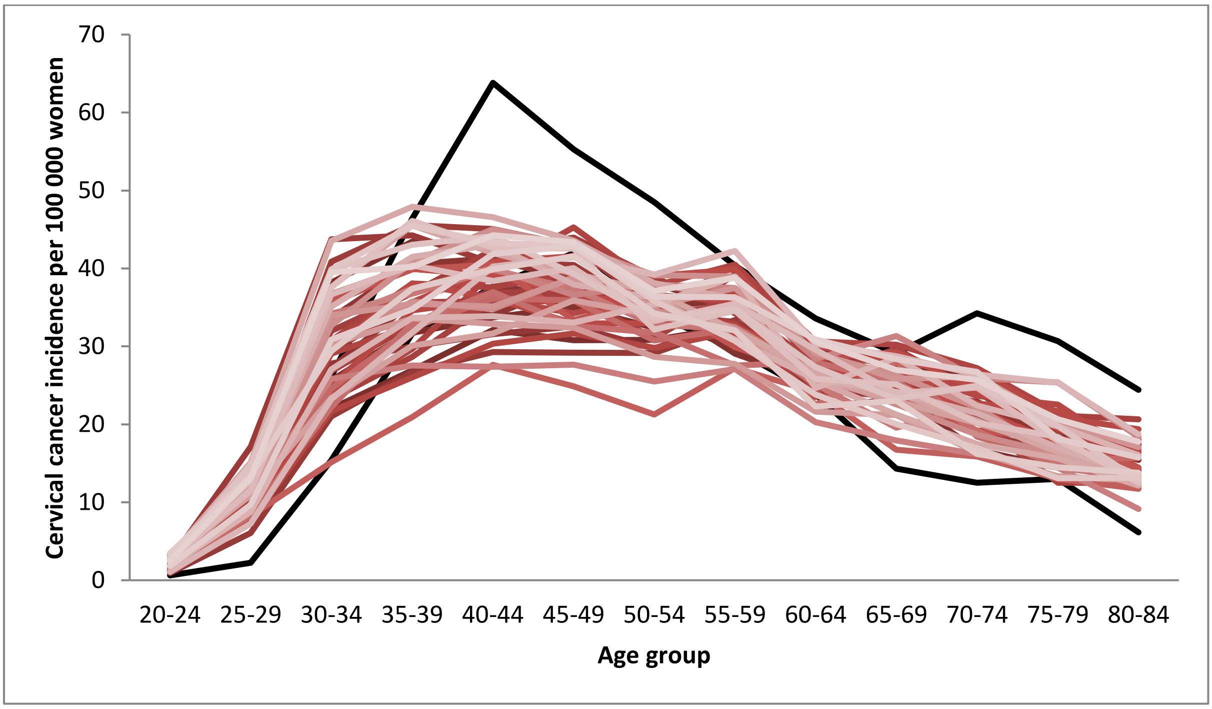

Current cervical cancer data were used to validate the natural history model. The primary data used were reported in a modified version of the FIGO system containing stages IA1-IVB and remapped to fit the FIGO system of local, regional and distant cancer.

Additional results

Figure S7: Discounted (3%) life expectancy and discounted costs per woman of 116 screening strategies under the baseline assumptions. including the 11 strategies on the efficiency frontier. The no screening strategy (natural history) has been excluded. Strategy nomenclature: “Start” stands for start age. y for years and HPV+/cyt- m stands for follow-up time for HPV-positive, cytology negative women (months).

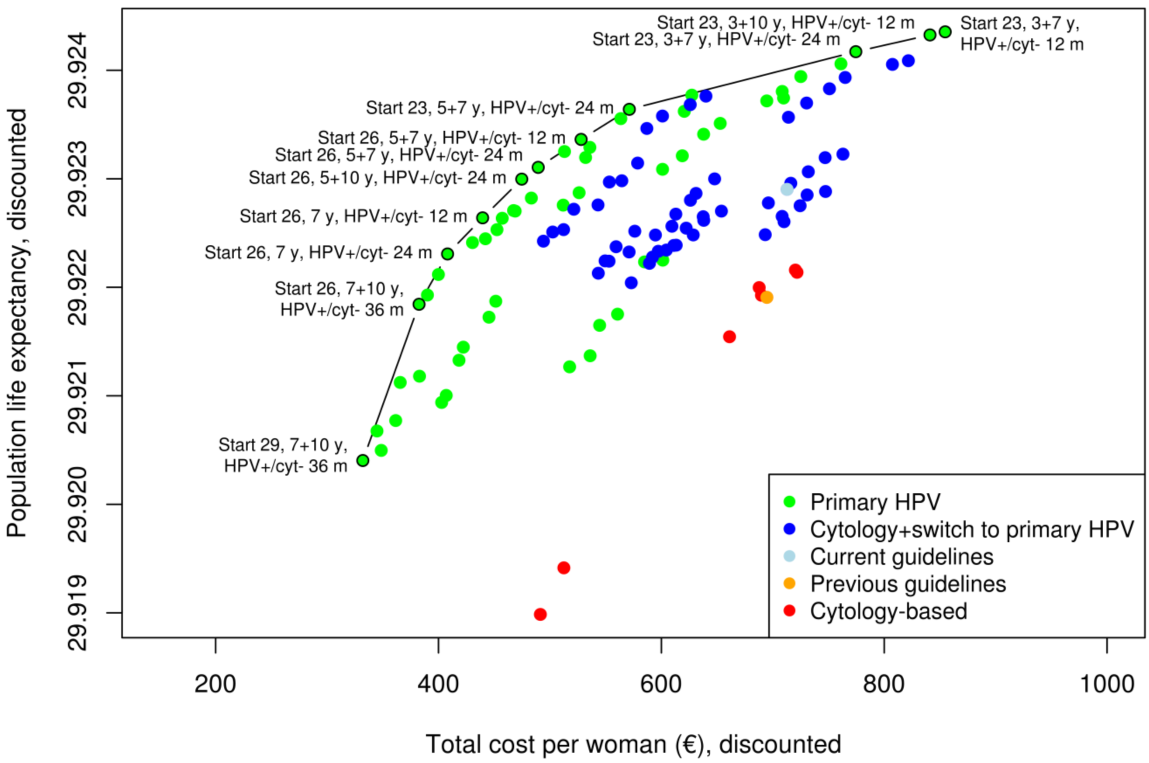

Table S4. Analytic outcomes for the no intervention scenario, the current and former Swedish guidelines, and strategies on the cost-efficiency frontier under a sensitivity analysis varying costs. The highlighted row represents the optimal strategy under the Swedish cost-effectiveness threshold.

Screening strategy characteristics						*Model predictions*					
**Screening regime**	**Cytology-based screening**	**Screening start age (years)**	**Screening interval (years) before age age 50**	**Screening interval (years) after age 50**	**HPV-pos/ Cyt-neg follow-up (months)**	**Lifetime cervical cancer risk**	**Proportion of cancers detected at a local stage**	**Expected no. of colposcopy referrals per woman over lifetime**	**Total lifetime cost per woman (discounted)**	**Discounted QALY**	**ICER (Cost per QALY gained)**
Natural History						1.73	0.510		193	26.53298	
Previous Program	Yes	23	3	5	-	0.28	0.646	0.479	508	26.57683	Dominated
Current Program	Before age 30	23	3	7	36	0.19	0.700	0.675	530	26.57775	Dominated
Primary HPV only	No	29	7	10	36	0.30	0.676	0.343	257	26.57397	1554
Primary HPV only	No	26	7	10	36	0.24	0.668	0.428	300	26.57635	17905
Primary HPV only	No	26	7	7	24	0.22	0.684	0.533	326	26.57696	43202
Primary HPV only	No	26	5	10	24	0.20	0.683	0.603	376	26.57780	59306
Primary HPV only	No	23	5	7	36	0.18	0.682	0.614	421	26.57847	67115
Primary HPV only	No	23	5	7	24	0.17	0.691	0.767	461	26.57892	88764
Primary HPV only	No	23	3	7	24	0.14	0.670	0.953	611	26.57968	196887
Primary HPV only	No	23	3	7	12	0.13	0.651	1.333	706	26.57999	306104

Abbreviations: CA cancer; HPV pos/Cyt neg HPV-positive, cytology-negative result; ICER, incremental cost-effectiveness ratio; QALY quality-adjusted life-years.

Table S5. Analytic outcomes for the no intervention scenario, the current and former Swedish guidelines, and strategies on the cost-efficiency frontier under a sensitivity analysis assuming HPV testing has 90 percent sensitivity (for all health states). The highlighted row represents the optimal strategy under the Swedish cost-effectiveness threshold.

Screening strategy characteristics						*Model predictions*					
**Screening regime**	**Cytology based**	**Start age**	**Screening interval (years)**	**Screening interval after 50 (years)**	**HPV pos/ Cyt neg follow-up (months)**	**Lifetime CA risk (%)**	**Proportion of localized CA**	**Expected no. of colposcopies per woman and lifetime**	**Total cost per woman (discounted)**	**Discounted QALY**	**QALY ICER**
Natural History						1.73	0.510		211	26.53276	
Previous Program	Yes	23	3	5	-	0.29	0.647	0. 456	692	26.57645	Dominated
Current Program	Before age 30	23	3	7	36	0.22	0.683	0. 627	708	26.57711	Dominated
Primary HPV only	No	29	7	10	36	0.37	0.653	0.298	332	26.57170	3118
Primary HPV only	No	26	7	10	36	0.31	0.649	0.373	379	26.57426	18562
Primary HPV only	No	26	7	10	24	0.30	0.650	0.451	394	26.57472	30673
Primary HPV only	No	26	7	7	24	0.28	0.662	0.460	401	26.57494	33170
Primary HPV only	No	26	5	10	24	0.25	0.661	0.522	465	26.57629	47585
Primary HPV only	No	23	5	7	24	0.21	0.667	0.666	556	26.57760	68850
Primary HPV only	No	23	3	7	24	0.17	0.663	0.835	756	26.57891	153130
Primary HPV only	No	23	3	10	12	0.16	0.646	1.137	811	26.57924	168149
Primary HPV only	No	23	3	7	12	0.16	0.642	1.155	824	26.57930	220533

Abbreviations: CA, cancer; HPV human papillomavirus; HPV pos/Cyt neg HPV positive, cytology negative; QALY quality adjusted life year; ICER incremental cost-effectiveness ratio.

Table S6. Analytic outcomes for the no intervention scenario, the current and former Swedish guidelines, and strategies on the cost-efficiency frontier under a sensitivity analysis assuming 10 percent of cervical cancers are HPV negative. The highlighted row represents the optimal strategy under the Swedish cost-effectiveness threshold.

Screening strategy characteristics						*Model predictions*					
**Screening regime**	**Cytology based**	**Start age**	**Screening interval (years)**	**Screening interval after 50 (years)**	**HPV-pos/ Cyt-neg follow-up (months)**	**Lifetime CA risk (%)**	**Proportion of localized CA**	**Expected no. of colposcopies per woman and lifetime**	**Total cost per woman (discounted)**	**Discounted QALY**	**QALY ICER**
Natural History						1.73	0.510		209	26.53307	
Previous Program	Yes	23	3	5	-	0.28	0.644	0.478	695	26.57681	Dominated
Current Program	Before age 30	23	3	7	36	0.19	0.693	0.674	713	26.57768	Dominated
Primary HPV only	No	29	7	10	36	0.30	0.662	0.343	332	26.57374	3016
Primary HPV only	No	26	7	10	36	0.24	0.657	0.427	382	26.57620	20461
Primary HPV only	No	26	7	10	24	0.23	0.662	0.523	400	26.57663	40631
Primary HPV only	No	26	7	7	24	0.22	0.671	0.533	408	26.57681	47465
Primary HPV only	No	23	7	10	36	0.21	0.643	0.530	442	26.57725	76643
Primary HPV only	No	23	5	10	24	0.17	0.673	0.759	564	26.57872	82716
Primary HPV only	No	23	5	7	24	0.17	0.678	0.767	571	26.57881	89545
Primary HPV only	No	23	5	7	12	0.16	0.659	1.043	627	26.57904	234724
Primary HPV only	No	23	3	10	12	0.13	0.649	1.311	841	26.57987	257976
Primary HPV only	No	23	3	7	12	0.13	0.640	1.332	854	26.57992	291132

Abbreviations: CA, cancer; HPV, human papillomavirus; HPV-pos/Cyt-neg, HPV-positive, cytology-negative; QALY, quality adjusted life year; ICER, incremental cost-effectiveness ratio.

Table S7. Analytic outcomes for the no intervention scenario, the current and former Swedish guidelines, and strategies on the cost-efficiency frontier under a sensitivity analysis varying compliance. The highlighted row represents the optimal strategy under the Swedish cost-effectiveness threshold

Screening strategy characteristics						*Model predictions*					
**Screening regime**	**Cytology based**	**Start age**	**Screening interval (years)**	**Screening interval after 50 (years)**	**HPV pos/ Cyt neg follow-up (months)**	**Lifetime CA risk (%)**	**Proportion of localized CA**	**Expected no. of colposcopies per woman and lifetime**	**Total cost per woman (discounted)**	**Discounted QALY**	**QALY ICER**
Natural History						1.73	0.510		210	26.53298	
Previous program	Yes	23	3	5	-	0.37	0.631	0.383	576	26.57407	Dominated
Current Program	Before age 30	23	3	7	36	0.28	0.668	0. 539	591	26.57497	Dominated
Primary HPV only	No	29	7	10	36	0.44	0.634	0.250	291	26.56933	2249
Primary HPV only	No	26	7	10	36	0.37	0.638	0.313	330	26.57211	13995
Primary HPV only	No	26	7	10	24	0.35	0.637	0.382	345	26.57295	17965
Primary HPV only	No	26	7	7	24	0.34	0.644	0.388	351	26.57313	28707
Primary HPV only	No	26	5	10	24	0.30	0.650	0.439	400	26.57478	30060
Primary HPV only	No	23	5	10	24	0.26	0.653	0.553	471	26.57638	44339
Primary HPV only	No	23	5	7	24	0.25	0.656	0.558	477	26.57647	56397
Primary HPV only	No	23	3	10	12	0.19	0.646	0.970	703	26.57865	103881
Primary HPV only	No	23	3	7	12	0.18	0.647	0.988	715	26.57876	111030

Abbreviations: CA, cancer; HPV human papillomavirus; HPV pos/Cyt neg HPV positive, cytology negative; QALY quality adjusted life year; ICER incremental cost-effectiveness ratio.

Table S8: Proportion of the 50 good-fitting parameter sets where scenarios from Table 2 where on the frontier. The highlighted row represents the optimal strategy under the Swedish cost-effectiveness threshold (base case analysis).

**Screening regime**	**Start age**	**Screening interval (years)**	**Screening interval after 50 (years)**	**HPV pos/ Cyt neg follow-up (months)**	**Proportion of the 50 sets where the scenario is on the frontier**
Primary HPV	29	7	10	36	1
Primary HPV	23	3	7	12	0.98
Primary HPV	26	7	10	36	0.94
**Primary HPV**	23	5	7	24	0.94
Primary HPV	23	3	10	12	0.76
Primary HPV	26	7	7	24	0.72
Primary HPV	26	7	10	24	0.6
Primary HPV	23	5	7	12	0.48
Primary HPV	26	5	10	24	0.42
Primary HPV	23	7	10	36	0.38
Primary HPV	23	5	10	24	0.36
Primary HPV	23	3	7	24	0.36
Primary HPV	23	5	7	36	0.24
Primary HPV	26	7	7	12	0.16
Primary HPV	26	5	10	12	0.16
Primary HPV	26	5	7	12	0.12
Primary HPV	23	7	10	24	0.1
Primary HPV	23	7	7	36	0.1
Primary HPV	26	5	7	24	0.1
Cytology with switch to primary HPV at age 35	23	5	7	12	0.08
Primary HPV	29	7	10	24	0.04
Primary HPV	23	3	10	24	0.02

References

Burger EA, Kim JJ, Sy S, Castle PE. Age of acquiring causal human papillomavirus (HPV) infections: Leveraging simulation models to explore the natural history of HPV-induced cervical cancer. Clin Infect Dis. 2017 Sep 15;65(6):893–9.

Campos NG, Burger EA, Sy S, Sharma M, Schiffman M, Rodriguez AC, et al. An updated natural history model of cervical cancer: derivation of model parameters. Am J Epidemiol. 2014 Sep 1;180(5):545–55.

Husereau D, Drummond M, Petrou S, Carswell C, Moher D, Greenberg D, et al. Consolidated Health Economic Evaluation Reporting Standards (CHEERS) statement. BMC Med. 2013 Mar 25;11:80.

Kim JJ, Campos NG, Sy S, Burger EA, Cuzick J, Castle PE, et al. Inefficiencies and high-value improvements in U.S. cervical cancer screening practice: A cost-effectiveness analysis. Ann Intern Med. 2015 Oct 20;163(8):589–97.

Olsen J, Jepsen MR. Human papillomavirus transmission and cost-effectiveness of introducing quadrivalent HPV vaccination in Denmark. International Journal of Technology Assessment in Health Care. 2010;26:183–191.

Pedersen K, Fogelberg S, Thamsborg LH, Clements M, Nygård M, Kristiansen IS, et al. An overview of cervical cancer epidemiology and prevention in Scandinavia. Acta Obstet Gynecol Scand. 2018 Jul;97(7):795–807.

Socialstyrelsen. Värdet av populationsbaserad screening för livmoderhalscancer. 2015. Socialstyrelsen, Stockholm.

Sparén P. Early detection and screening for cancer of the cervix in Sweden during the 20th century. 1996. PhD thesis. Uppsala University.

Östensson E, Fröberg M, Leval A, Hellström A-C, Bäcklund M, Zethraeus N, et al. Cost of preventing, managing, and treating human papillomavirus (HPV)-related diseases in Sweden before the introduction of quadrivalent HPV vaccination. PLoS ONE. 2015;10(9):e0139062.
